# Supplementary material for: Who seeks care after intimate partner violence in Cameroon? sociodemographic differences between a hospital and population sample of women
Source: PLOS Glob Public Health. 2024 Jul 19;4(7):e0003408. doi: 10.1371/journal.pgph.0003408 (PMC11259300; doi:10.1371/journal.pgph.0003408)
Supplement: S1 Table — While the concatenated CTR and DHS databases had several similarities between variables, each database defined certain variables slightly differently. This table explains the differences in variables and highlights how the variables were considered during data analysis. CTR = Cameroon Trauma Registry, DHS = Demographic and Health Survey, LPG = liquid petroleum gas. (DOCX) [file pgph.0003408.s002.docx]

**S1 Table. Comparison of variables in CTR and DHS datasets.**

While the concatenated CTR and DHS databases had several similarities between variables, each database defined certain variables slightly differently. This table explains the differences in variables and highlights how the variables were considered during data analysis.

| Variable | CTR | DHS |
| --- | --- | --- |
| Age | Asked participants their age. Collected as a continuous variable and categorized for analysis. | Same |
| Urban residence | Asked participants if they lived in urban or rural residence | Same |
| Cellphone ownership | Asked if household owned a mobile or cellular phone. | Same |
| Employment | Asked if individuals were employed in a salaried or self-employed position, without a time reference. For analysis, either employment response was considered as “employed.” | Asked if respondent had worked in in the past year. Considered respondents employed if they affirmed that they worked in the past year or if they indicated they had a job but were on leave for the past seven days. |
| Education | Asked the highest year of study attempted (regardless of completion). The highest level of education is university. Analysis examined highest level of education attempted. | Asked the highest year of study and whether the level of education is complete or incomplete. The highest level as “higher than secondary.” |
| Ownership of agricultural land | Asked if household owns agricultural land. | Asked whether household “owns land usable for agriculture.” |
| Home ownership | Asked if household owns, rents, or lives for free in their residence. Considered to own home if respondent indicated they “own” their residence. | Asked if respondent owns home jointly, alone, or both jointly and alone. Considered to own home if respondent affirmed they owned home, regardless of alone/jointly. |
| Cooking fuel | Asked all types of cooking fuel used in household. Variable was categorized into a discrete choice variable based on the most expensive cooking fuel indicated. For analysis, we categorized cooking fuel as LPG and non-LPG. | Asked respondents to report one type of cooking fuel most commonly used by the household. |

Legend: CTR = Cameroon Trauma Registry, DHS = Demographic and Health Survey, LPG = liquid petroleum gas
