# Supplementary material for: Who seeks care after intimate partner violence in Cameroon? sociodemographic differences between a hospital and population sample of women
Source: PLOS Glob Public Health. 2024 Jul 19;4(7):e0003408. doi: 10.1371/journal.pgph.0003408 (PMC11259300; doi:10.1371/journal.pgph.0003408)
Supplement: Attachment — Submitted filename: Response to Reviewers.docx [file pgph.0003408.s004.docx]

*PLOS Global Public Health*

Response to Reviewer Comments

Received 22 January 2024

Due 21 February 2024

Dear *PLOS Global Public Health* Reviewers and Editors,

Thank you very much for your comments and suggested edits. We greatly appreciate the opportunity to improve the manuscript. Please see our responses to comments below. Please note that the reviewer and editor comments are in *italics* while our responses to comments are not italicized.

===

**Journal Requirements:**

1. *You indicated that you had ethical approval for your study. In your Methods section, please ensure you have also stated whether you obtained consent from parents or guardians of the minors included in the study or whether the research ethics committee or IRB specifically waived the need for their consent.*

Thank you for your comment. For minors enrolled in the Cameroon Trauma Registry (CTR), research assistants obtained consent from parents and/or guardians of the minors. Edits have been made in the “Ethics” subsection of the Methods section.

2. *Please include a complete copy of PLOS’ questionnaire on inclusivity in global research in your revised manuscript. Our policy for research in this area aims to improve transparency in the reporting of research performed outside of researchers’ own country or community. The policy applies to researchers who have travelled to a different country to conduct research, research with Indigenous populations or their lands, and research on cultural artefacts. The questionnaire can also be requested at the journal’s discretion for any other submissions, even if these conditions are not met.  Please find more information on the policy and a link to download a blank copy of the questionnaire here:* [*https://journals.plos.org/globalpublichealth/s/best-practices-in-research-reporting*](https://journals.plos.org/globalpublichealth/s/best-practices-in-research-reporting)*. Please upload a completed version of your questionnaire as Supporting Information when you resubmit your manuscript*.

Thank you for your comment. We have uploaded the questionnaire as Supporting Information.

*Additional Editor Comments (if provided): None*

**Reviewers' comments:
Reviewer's Responses to Questions**

***Comments to the Author***

1. *Does this manuscript meet PLOS Global Public Health’s* [*publication criteria*](https://journals.plos.org/globalpublichealth/s/criteria-for-publication)*? Is the manuscript technically sound, and do the data support the conclusions? The manuscript must describe methodologically and ethically rigorous research with conclusions that are appropriately drawn based on the data presented.*
   a. *Reviewer #1: Yes*
   b. *Reviewer #2: Yes*
2. *Has the statistical analysis been performed appropriately and rigorously?*
   a. *Reviewer #1: Yes*
   b. *Reviewer #2: Yes*
3. *Have the authors made all data underlying the findings in their manuscript fully available (please refer to the Data Availability Statement at the start of the manuscript PDF file)? The* [*PLOS Data policy*](https://journals.plos.org/globalpublichealth/s/data-availability) *requires authors to make all data underlying the findings described in their manuscript fully available without restriction, with rare exception. The data should be provided as part of the manuscript or its supporting information, or deposited to a public repository. For example, in addition to summary statistics, the data points behind means, medians and variance measures should be available. If there are restrictions on publicly sharing data—e.g. participant privacy or use of data from a third party—those must be specified.*
   a. *Reviewer #1: Yes*
   b. *Reviewer #2: Yes*
4. *Is the manuscript presented in an intelligible fashion and written in standard English? PLOS Global Public Health does not copyedit accepted manuscripts, so the language in submitted articles must be clear, correct, and unambiguous. Any typographical or grammatical errors should be corrected at revision, so please note any specific errors here.*
   a. *Reviewer #1: Yes*
   b. *Reviewer #2: Yes*
5. *Review Comments to the Author. Please use the space provided to explain your answers to the questions above. You may also include additional comments for the author, including concerns about dual publication, research ethics, or publication ethics. (Please upload your review as an attachment if it exceeds 20,000 characters)*
   a. *Reviewer #1: I think PLOS criteria has been met, but the larger issue of VAW needs to be acknowledged and how this study contributes to the larger goal could be included.*

Thank you for your comment. We agree that the large issue of violence against women needs to be acknowledged. We added additional information to the first paragraph of the Discussion section to further elaborate on this issue.

b. *Reviewer #1: Additional comments: a para could be added in the introduction explaining why interviews with survivors were not conducted, and why it is important to interview them. The paper mentions that qualitative approach was not taken but does not explain why it could not be. this gap should be stated clearly stated. Mention of stakeholders for VAW mitigation could be included and a dissemination plan for reaching them outlined.*

Thank you for your comment. We agree that a qualitative study examining the care seeking behaviors of women who experience violence would provide richer data and accentuate our study findings. While qualitative methods were outside the scope of this cross-sectional quantitative study, we included additional information in the final paragraph of the Discussion section of this manuscript to highlight the importance of qualitative methods on future violence against women research and interventions.

We included additional information in the final paragraph in the Discussion section of this manuscript to explain the impact of qualitative methods on future violence against women research and interventions.

c. *Reviewer #2: The paper was adequately motivated and no doubt has a valid research gap which has been addressed. However, there are extant literature on IPV that should be reviewed to provide a basis for the hypothesis "that women with higher SES and education levels would be more likely to present to a hospital for care of injuries sustained from IPV".*

Thank you for your comment. We have modified the language regarding the hypothesis. Rather than writing “higher SES,” we have clarified the language to say “women of a higher socioeconomic position” to better reflect the literature term that describes women of higher SES and higher education levels. We have included references and additional sentences to the last paragraph of the Introduction section to provide an evidence basis and further rationale for the hypothesis.

d. *Reviewer #2: Also, at best the findings are mere "associations". Whether policy implication can be drawn from such findings is unclear. This should interest stakeholders.*

Thank you for your comment. We agree that our study methods methodology cannot establish causative relationships between variables and outcomes. Rather, our statistical methods seek to find associations between variables and outcomes of interest. We added language to the limitations paragraph in the Discussion section to address these noted limitations.

e. *Reviewer #2: Additionally, on lines 107-108 “ We excluded women from households in other regions of Cameroon” Can they authors provide rationale for the exclusion? And other similar eligibility criteria used in the selection of the final sample?*

Thank you for your comment. During the study period, the CTR collected data at four hospitals in two regions of Cameroon, the Southwest and Littoral regions. Cameroon has ten regions that govern semi-autonomously (i.e., the regional governments function somewhat similarly as state governments function in the United States). The 2018 Demographic and Health Survey (DHS) was a national survey conducted in all regions throughout Cameroon.

To make the DHS cohort as similar as possible to the CTR cohort in our study, we only included DHS responses from women who lived in the same regions as the CTR hospitals – the Southwest and Littoral regions. Please note that the DHS dataset categorized Douala as its own region, although it is the capital city of the Littoral region. Thus, we included the DHS dataset “regions” of Douala, Littoral, and Southwest in our DHS cohort analysis. We excluded women from the eight other regions to avoid any underlying regional differences that may confound study findings.

We have added additional description in the “Setting” subsection of the Methods section to better describe the inclusion criteria and reasoning behind it.

f. *R*evie*wer #2: Further, on lines 142-143 “Any missing observations on the outcome and predictor variables underwent listwise deletion in the regression”. Can they authors mention such variables and how "significant" were their omission?*

Thank you for your comment. Stata, the statistical software that we used for regression analysis, automatically performs listwise deletion of rows that contain missing data in the variable of interest.

As shown in Table 3, our regression model included age group, ownership of agricultural land, attainment of university-level education, and use of liquid petroleum gas (LPG) cooking fuel. Missing data for each variable is shown in Table 2. Since less than 10% of data was missing from each included variable, we do not believe their omission to be significant. Moreover, we did not want to create separate dummy variable categories for missing data since data missingness was only present in the CTR dataset and the number of observations were so few.

We have edited the “Statistical methods and data analysis” subsection of the Methods section and the Discussion section to elaborate further on this methodology.

g. *Reviewer #2: Finally, does the "conclusion that Women who experienced violence presenting to the hospital have characteristics associated with higher SES and are less likely to demonstrate factors associated with residence in rural setting compared to the general population of women experiencing violence" free from self selection bias? Could factor such as exposure to mass media confound the findings?*

Thank you for your comment. We agree that selection bias is present in this study. We do not believe exposure to mass media to be a significant confound since most people in Cameroon have access to modern technology such as cellphones. We have edited the limitations paragraph in the Discussion section to address these concerns.

6. *PLOS authors have the option to publish the peer review history of their article (*[*what does this mean?*](https://journals.plos.org/globalpublichealth/s/editorial-and-peer-review-process#loc-peer-review-history)*). If published, this will include your full peer review and any attached files.* ***Do you want your identity to be public for this peer review?*** *If you choose “no”, your identity will remain anonymous but your review may still be made public. For information about this choice, including consent withdrawal, please see our* [*Privacy Policy*](https://www.plos.org/privacy-policy)*.*
   a. *Reviewer #1: No*
   b. *Reviewer #2:* ***Yes:*** *Atsiya PIUS AMOS*
