# Supplementary material for: Who seeks care after intimate partner violence in Cameroon? sociodemographic differences between a hospital and population sample of women
Source: PLOS Glob Public Health. 2024 Jul 19;4(7):e0003408. doi: 10.1371/journal.pgph.0003408 (PMC11259300; doi:10.1371/journal.pgph.0003408)
Supplement: Attachment — Submitted filename: Response to Reviewers_R2.docx [file pgph.0003408.s005.docx]

*PLOS Global Public Health*

Response to Reviewer Comments #2

Received 10 May 2024

Submitted 13 May 2024

Dear *PLOS Global Public Health* Reviewers and Editors,

Thank you very much for your comments and suggested edits. We greatly appreciate the opportunity to improve the manuscript. Please see our responses to comments below. Please note that the reviewer and editor comments are in *italics* while our responses to comments are not italicized.

===

**Journal Requirements:**

1. *You indicated that you had ethical approval for your study. In your Methods section, please ensure you have also stated whether you obtained consent from parents or guardians of the minors included in the study or whether the research ethics committee or IRB specifically waived the need for their consent.*

We have stated that research assistants obtained consent from parents and/or guardians of the minors in the “Ethics” subsection of the Methods section.

2. *Please include a complete copy of PLOS’ questionnaire on inclusivity in global research in your revised manuscript. Our policy for research in this area aims to improve transparency in the reporting of research performed outside of researchers’ own country or community. The policy applies to researchers who have travelled to a different country to conduct research, research with Indigenous populations or their lands, and research on cultural artefacts. The questionnaire can also be requested at the journal’s discretion for any other submissions, even if these conditions are not met.  Please find more information on the policy and a link to download a blank copy of the questionnaire here:* [*https://journals.plos.org/globalpublichealth/s/best-practices-in-research-reporting*](https://journals.plos.org/globalpublichealth/s/best-practices-in-research-reporting)*. Please upload a completed version of your questionnaire as Supporting Information when you resubmit your manuscript*.

We have uploaded the questionnaire as Supporting Information.

3. *Please review your reference list to ensure that it is complete and correct. If you have cited papers that have been retracted, please include the rationale for doing so in the manuscript text, or remove these references and replace them with relevant current references. Any changes to the reference list should be mentioned in the rebuttal letter that accompanies your revised manuscript. If you need to cite a retracted article, indicate the article’s retracted status in the References list and also include a citation and full reference for the retraction notice.*

All references have been reviewed and the reference list is complete and correct. There are no citations of papers that have been retracted. Based on the comments from Reviewer #3, we have added two additions to the references (Reference #36 and #39).

*Additional Editor Comments (if provided): None*

**Reviewers' comments:
Reviewer's Responses to Questions**

***Comments to the Author***

1. *If the authors have adequately addressed your comments raised in a previous round of review and you feel that this manuscript is now acceptable for publication, you may indicate that here to bypass the “Comments to the Author” section, enter your conflict of interest statement in the “Confidential to Editor” section, and submit your "Accept" recommendation.*
   a. *Reviewer #3: (No Response)*
2. *Does this manuscript meet PLOS Global Public Health’s* [*publication criteria*](https://journals.plos.org/globalpublichealth/s/criteria-for-publication)*? Is the manuscript technically sound, and do the data support the conclusions? The manuscript must describe methodologically and ethically rigorous research with conclusions that are appropriately drawn based on the data presented.*
   a. *Reviewer #3: Yes*
3. Has the statistical analysis been performed appropriately and rigorously?
   a. *Reviewer #3: Yes*
4. *Have the authors made all data underlying the findings in their manuscript fully available (please refer to the Data Availability Statement at the start of the manuscript PDF file)? The* [*PLOS Data policy*](https://journals.plos.org/globalpublichealth/s/data-availability) *requires authors to make all data underlying the findings described in their manuscript fully available without restriction, with rare exception. The data should be provided as part of the manuscript or its supporting information, or deposited to a public repository. For example, in addition to summary statistics, the data points behind means, medians and variance measures should be available. If there are restrictions on publicly sharing data—e.g. participant privacy or use of data from a third party—those must be specified.*
   a. *Reviewer #3: Yes*
5. *Is the manuscript presented in an intelligible fashion and written in standard English? PLOS Global Public Health does not copyedit accepted manuscripts, so the language in submitted articles must be clear, correct, and unambiguous. Any typographical or grammatical errors should be corrected at revision, so please note any specific errors here.*
   a. *Reviewer #3: Yes*
6. *Review Comments to the Author. Please use the space provided to explain your answers to the questions above. You may also include additional comments for the author, including concerns about dual publication, research ethics, or publication ethics. (Please upload your review as an attachment if it exceeds 20,000 characters)*
   a. *Reviewer #3: First of all, I would like to congratulate you for the work you have done and in particular for trying to focus on a problem of global magnitude such as violence against women, specifically Intimate Partner Violence (IPV). Unfortunately, in the context of the study, the approach taken is one of the possible ones, although it is the tip of the iceberg of the problem. The study is important in itself because of what it shows, but even more so because of what it is not possible to show. I think the authors are aware of the latter, and it would be of interest if it were shown in the article.*

Thank you for your comment. We agree that there are many anecdotal findings relating to IPV that our research group knows that are not possible to show in this study. Since we performed a quantitative analysis, we wanted this manuscript to focus on the quantitative findings present in the data. We wanted to avoid anecdotal commentary that may be construed as by the audience as speculative given the quantitative nature of the manuscript. Nevertheless, we incorporated your comments below to advocate in the Discussion and Conclusion sections for an expansion of future study into IPV.

b. *Reviewer #3: I will make specific comments on the following article: Methods. Explain on the basis of which you have chosen the 15-49 age range, and what this decision entails. On the one hand, you include minors, 15-18 years old, young women, 18-35 years old, and adult women, 35-49 years old. The circumstances of minor women will be different from those of young or adult women. In this regard, I think it is important to explain why this decision was made, and to think about whether to add additional information where appropriate in this regard. It would be desirable to find information on this in the limitations section of the study.*

Thank you for your comment. We chose the 15-49 age range to best match the samples present in both the CTR and DHS databases. Specifically, the DHS reports findings regarding domestic violence among women using the age range of 15-49 years. We have made relevant edits in the “Participants and Data Sources” subsection in the Methods section of the paper to address this point.

This age range is consistently used in other studies, we referenced an additional study that uses the 15-49 age range in the Discussion section of the paper. We agree with the reviewer comment that at 15-year-old woman will have a different demographic and risk factor profile compared to a 49-year-old woman. However, any sub-group analysis of specific age groups in our study was not possible due to small sample size. We have made relevant edits in limitations paragraph in the Discussion section of the paper.

c. *Reviewer #3: Results and discussion. Incorporate a bibliographic reference that contextualizes in depth the importance of the LPG indicator. I suggest a bibliographic reference, by way of example, but consider incorporating a more appropriate one. Household Determinants of Liquified Petroleum Gas (LPG) as a Cooking Fuel in South West Cameroon. Pope D, Bruce N, Higgerson J, Hyseni L, Ronzi S, Stanistreet D, MBatchou B, Puzzolo E. Ecohealth. 2018 Dec;15(4):729-743. doi: 10.1007/s10393-018-1367-9. Epub 2018 Oct 1.*

Thank you for the suggested reference. We have reviewed this article and included it as a reference in the Discussion section of the manuscript.

d. *Reviewer #3: It is positive that you are providing information in relation to new lines of research. Specifically, they refer to research in which qualitative methodology would be used. In this case, I suggest that you do not only talk about a qualitative research technique, such as the interview, but also allude to the importance of seeking answers to questions that can be answered using another type of methodology, such as qualitative. Indeed, collecting qualitative information among women who have suffered IPV, health professionals, state security forces, social workers, workers of non-governmental organizations, and even, as you say, among the perpetrators of IPV, would be essential to gain an in-depth understanding of the problem you are addressing. It would also be interesting to comment on the need to develop research to know the social perceptions of the population of Cameroon in relation to IPV, as well as studies aimed at raising awareness among the population in relation to this social and public health problem. Personally, I encourage you to develop these future lines of research.*

Thank you for your comment. We agree that the complexity of IPV requires multiple research perspectives to best describe the problem and develop context-specific IPV mitigation interventions. We have edited the Discussion section of the manuscript to comment on the importance of studying social perceptions of IPV in Cameroon. Additionally, we have also added comments about the importance raising awareness of IPV among the population through dissemination of findings.

Finally, we agree that the public health problem of IPV requires further study and intervention to ultimately, lessen the burden of injury due to IPV in Cameroon. We hope that the publication of this study will serve as a first step for our research group to conduct further IPV interventions over time. We aim to use this evidence base as preliminary findings for future, larger scale studies of IPV intervention.

e. *Reviewer #3: Conclusions. I suggest that the final sentences of the conclusions be along the lines of reinforcing issues raised in the manuscript, beyond the specific study, in relation to the need to develop more research studies on IPV in the context of Cameroon, in order to gain an in-depth understanding of this problem, which is a global problem.*

Thank you for your comment. We have added additional text in the Conclusion section of the manuscript that advocates for more research of IPV in the context of Cameroon.

7. *PLOS authors have the option to publish the peer review history of their article (*[*what does this mean?*](https://journals.plos.org/globalpublichealth/s/editorial-and-peer-review-process#loc-peer-review-history)*). If published, this will include your full peer review and any attached files.* ***Do you want your identity to be public for this peer review?*** *If you choose “no”, your identity will remain anonymous but your review may still be made public. For information about this choice, including consent withdrawal, please see our* [*Privacy Policy*](https://www.plos.org/privacy-policy)*.*
   a. *Reviewer #3: No*
